# Production of *Trans*‐free fats by chemical interesterified blends of palm stearin and sunflower oil

**DOI:** 10.1002/fsn3.1231

**Published:** 2019-10-03

**Authors:** Davood Farajzadeh Alan, Mohammad Hossein Naeli, Mehdi Naderi, Seid Mahdi Jafari, Hamid Reza Tavakoli

**Affiliations:** ^1^ Medicine, Quran and Hadith Research Center Baqiyatallah University of Medical Sciences Tehran Iran; ^2^ Department of Food Science and Technology Faculty of Agricultural Engineering Sari Agricultural Sciences and Natural Resources University Sari Iran; ^3^ Department of Food Materials and Process Design Engineering Faculty of Food Science and Technology Gorgan University of Agricultural Sciences and Natural Resources Gorgan Iran; ^4^ Health Research Center Life Style Institute Baqiyatallah University of medical Sciences Tehran Iran

**Keywords:** chemical interesterification, palm stearin, production, sunflower oil, *Trans*‐free fats

## Abstract

In this study, production of *trans*‐free fats through chemical interesterification of binary blends of palm stearin (PS) and sunflower oil (SFO) and their physicochemical changes after the process was investigated. Analyzed responses included fatty acid and triacylglycerol composition, iodine value, free fatty acid (FFA), soap content, peroxide value (PV), plastic range, slip melting point (SMP), solid fat content (SFC), and oxidative stability along with potential applications of the interesterified fats. *Trans*fatty acid content of PS/SFO blends was lower than 0.36%. Chemical interesterification increased the FFA and soap content and also decreased PV and oxidative stability index (at 110°C). After the process, SMP and SFC were reduced, also the plastic range transferred to the lower temperatures. All the interesterified blends melted completely at the body temperature, and their SFC was <32%. The melting characteristics of the PS/SFO‐interesterified blends were suitable for many fat‐based products.

## 1 INTRODUCTION

Oils and fats are one of the important nutrients in the human diet and have a key role in improving the palatability of foods (Fauzi, Rashid, & Omar, 2013; Jalili, Jafari, Emam‐Djomeh, Malekjani, & Farzaneh, 2018). In the food industry, bakery (shortening) and spreadable (margarine) fats impart important favorable functional and organoleptic properties such as spreadability, consistency stability, lubricity, tenderness and texture, aeration, heat transfer, desirable favor, and taste, positively contributing to the structure and geometry of the final products. In this regard, these fats must have special physicochemical properties including a desirable melting behavior, consistency, and plasticity as well as thermal and oxidative stability. Regarding the fat base of margarine and shortening, functionality, formulation, production procedure, and cost efficiency as well as nutritional specifications should be considered to meet consumer demands on public health (Saghafi, Naeli, Bahmaei, Tabibiazar, & Zargaraan, 2019).

The main disadvantage of vegetable oils and fats is their limited application in natural state due to their specific chemical composition (Taghvaei & Jafari, 2015; Taghvaei, Jafari, Nowrouzieh, & Alishah, 2013). Most oils and fats have a certain distribution pattern of fatty acids on the glycerol backbone, which affects their physicochemical properties and applications (Karabulut, Turan, & Ergin, 2004). In order to modify the functional features of oils and fats, chemical processes such as hydrogenation and interesterification or physical processes such as fractionation are being applied (Fauzi et al., 2013). Although partial hydrogenation is one of the most practical and common methods to modify oils and fats, it produces isomer *trans* (da Silva et al., 2010). There is a direct relationship between intakes of *trans*fatty acid (TFA) and increased risk for coronary heart diseases. In this context, it is necessary to address the food safety problems caused by TFAs, which are mainly produced by hydrogenation. Therefore, reduction of TFAs in food products has become one of the challenges and tasks of the food industry professionals (Ahmadi & Marangoni, 2009).

Chemical interesterification is a promising technique for modifying the physicochemical properties of lipids with various industrial applications, without production of isomer *trans*. Contrary to hydrogenation process, interesterification does not affect the SFA and also TFA. If *trans*‐free fats could be used as the base stocks of interesterification, interesterified (IE) fats also will be *trans*‐free (Farmani, Safari, & Hamedi, 2009; Naeli, Farmani, & Zargaraan, 2017). During chemical interesterification, fatty acids are exchanged with a random template on the glycerol groups until a thermodynamic equilibrium is achieved (Farmani et al., 2009; Naeli, Farmani, & Zargaraan, 2018; da Silva et al., 2010). Unlike blending, chemical interesterification can lead to the increased consistency within the solid phase and also will improve the plasticity of fat. Furthermore, this process can either increase or decrease the melting point and solid fat content (SFC) of fat blends depending on the initial triacylglycerol (TAG) composition of fat blend (Costales‐Rodríguez, Gibon, Verhé, & De Greyt, 2009). Behmadi, Zandi, Goldani, and Ghavami (2008) evaluated the influence of chemical interesterification on the blend of sunflower oil and fully hydrogenated soybean oil (70/30) to produce a tailor‐made fat. They found reduction in the slip melt point (from 56 to 34°C).

Structured fats are suitable for product such as margarines and shortenings. Zandi , Goldani, Behmadi, Khoushtinat, & Hosseini (2003) investigated the possibility of random chemical interesterification in pilot scale by using cottonseed, sunflower and soybean oils (as liquid phase), and fully hydrogenated soybean oil (as solid phase); they reported a significant decrease in slip melting point (SMP, from 54 to 35°C). Other results have also showed that interesterification is capable of decreasing SFC, so that the SFC curve is presenting more proper plastic properties within the product. Fauzi et al. (2013) set up a study on the mixture of palm stearin (PS), palm kernel oil, and soybean oil which were subjected to chemical interesterification. They reported that changes in the physicochemical properties of mentioned modified oils are a result of chemical interesterification.

Palm oil fractions (palm olein and PS) are becoming main base stocks in producing margarines and shortenings. The recent interest in removing the TFA from margarines and shortenings has been focused on palm oil derivatives which are the main source of natural solid fats. PS is a solid fraction which is physically derived by fractionation of palm oil without any hydrogenation process. Unfortunately, PS is not used directly for edible purposes because it has a high melting point and a low plasticity. Therefore, interesterification or blending with other oils is applied to improve melting profiles of PS (Andreia Schäfer De Martini Soares et al., 2009; Dian, Sundram, & Idris, 2007).

On the other hand, sunflower oil (SFO) is one of the most widely used vegetable oils in the world, possibly because sunflower is one of the most widely cultivated and utilized plants, particularly in recent decades. It is rich in ω_9_ and ω_6_ fatty acids (Kummerow, Mahfouz, & Zhou, 2007; Noori et al., 2011). Accordingly, production of plastic fats from PS and SFO is interesting.

In the present study, for production of zero *trans*plastic fats, binary blends of PS: SFO in ratios (w/w) of 10:90, 30:70, and 50:50 were chemically interesterified and some physicochemical properties of the obtained fats were investigated. Finally, the potential application of interesterified blends in the production of various types of fats was also studied. The main aim of this work was to investigate the effect of chemical interesterification on the fatty acid composition, free fatty acids, soap content and peroxide value, slip melting point (SMP), solid fat content (SFC), plastic range, and oxidative stability index of PS: SFO blends. Practically, the presented *trans*‐free fats can be used as a base stock for many fatty product formulations.

## 2 MATERIALS AND METHODS

Refined, bleached, and deodorized SFO and PS were obtained from Kourosh Co. The dry powder of sodium methoxide was purchased from Merck Co. Other chemicals were of analytical grade and purchased from Merck.

### 2.1 Chemical interesterification of PS/SFO

At first, PS was melted at 85°C and blends of PS:SFO in the mass ratios of 10:90, 30:70, and 50:50 were prepared (400 g). Prior to chemical interesterification, the blends were heated under vacuum (at 0.8 bar abs, 100°C for 15 min) to remove traces of water (Farmani et al., 2009). Dried blends were charged into a vacuum flask and brought to 90°C. In the next step, 0.5% (w/w) dry sodium methoxide was added into the dried fat blends, and the interesterification process continued for 1 hr (at 90°C, under 0.8 bar abs and 300 rpm). During the interesterification process, SMP of fat blends alters until reaching constant values. Therefore, the reaction equilibrium was monitored by SMP determination. After reaching the reaction equilibrium point, to inactivate the catalyst, 2% (w/w) aqueous citric acid solution (20%, w/v) was added. Then, the stirring of mixture was continued for 15 min (at 70°C and 300 rpm). The excess alkalinity, sodium methoxide, and citric acid were removed by addition of 1.5% bleaching earth (bentonite) into the blends (at 300 rpm, under 0.8 bar abs and at 110°C for 15 min). Finally, the bleaching earth was separated from the mixture of fat by filter aid‐settled and Whatman filter paper, grade 4, twice (Saghafi, Naeli, Tabibiazar, & Zargaraan, 2018).

### 2.2 Determination of fatty acid composition

Identification and quantification of fatty acid composition of the interesterified samples was analyzed by Agilent Acme 6100 gas chromatograph equipped with a flame ionization detector, according to the American Oil Chemists' Society (AOCS) methods Ce 2‐66 and Ce 1‐91 (AOCS, 1996). Nitrogen was the carrier gas, and the column head pressure was 29.5 psi. The detector and injector temperature were 280 and 240°C, respectively. The capillary chromatographic column CP Sil 88 (100 m, 0.25 mm inner diameter and 0.25 μm film thickness) was used to analyze the fatty acid methyl esters with 1:40 split ratio.

### 2.3 Analysis of iodine value

Iodine value was measured using AOCS method Cd 1c‐85 (AOCS, 1996) according to Equation 1 and considering the contents of oleic acid (C18:1), linoleic acid (C18:2), and linolenic acid (C18:3).

(1) $$\text{Iodine}\;\text{value}=\left(\%\;C18:1\times 0.89\right)+\left(\%\;C18:2\times 1.732\right)+\left(\%\;C18:3\times 2.616\right)$$

### 2.4 Calculation of triacylglycerol composition (TAG)

Triacylglycerol compositions of PS and SFO were obtained from the literature (Costales‐Rodríguez et al., 2009; Lida, Sundram, Siew, Aminah, & Mamot, 2002) and used for the calculation of the TAG composition of NIE blends. Also, TAG composition of IE blends was calculated based on the 1, 2, 3‐random theory following the probability law from the fatty acid composition.

### 2.5 Determination of free fatty acids, soap content, and peroxide value

Free fatty acid (FFA), soap content, and peroxide value (PV) were evaluated according to AOCS Cd 8‐53, AOCS Cc 17‐95, and AOCS Ca 5a‐40 methods, respectively (AOCS, 1996).

### 2.6 Analysis of slip melting point (SMP)

The SMP of blends was analyzed in accordance with AOCS Cc 3‐25 open tube melting point after tempering at 6 ± 1°C for 16 hr (AOCS, 1996).

### 2.7 Measurement of solid fat content (SFC)

Solid fat content (5, 10, 20, 30, 40, 45, and 50°C) was determined using a minispec mq 20 pulsed nuclear magnetic resonance (NMR) spectroscope (Bruker Corporation), according to the AOCS method Cd 16b‐93, direct serial measurement method (AOCS, 1996). Before the first SFC measurement, the NMR tubes filled with melted fat samples were placed in an ice‐bath (0°C) for 60 min. Following, the sample tubes were conditioned in desired temperatures (5, 10, 20, 30, 40, 45, and 50°C) for 35 min, and then, the SFC was read at each temperature.

### 2.8 Determination of plastic range

Plastic range is a temperature range in which fats have a good consistency which can be obtained by measuring the SFC in the range of 15%–25%. For this purpose, the sigmoidal Gompertz function was used to fit the SFC‐temperature curve of each blend before and after interesterification. The function is described in Equation 2, in which, *α* is the upper asymptote, *b* sets the ordinate axis displacement, *c* sets the growth rate (Y scaling) and *T* is temperature. The parameters of the Gompertz function (*α*,* b*, and *c*) were calculated for each SFC‐temperature curve using SigmaPlot software ver. 12 (Systat Software Inc.). Then, the plastic range was determined by the substitution of known SFCs (15%–25%) in the Equation 2 (Naeli et al., 2017).

(2) $$\text{SFC}=\alpha e^{-e^{-\left(\frac{T-c}{b}\right)}}$$

### 2.9 Analysis of oxidative stability index

To measure induction periods (IP) of oxidation, according to the AOCS method Cd 12b‐92, a Metrohm Rancimat instrument model 743 was used (with 2.5 g sample, at 110°C, and air flow rate of 2.5 ml/s; AOCS, 1996).

### 2.10 Oxidizability measurement

Based on the contents of oleic acid (C18:1), linoleic acid (C18:2), and linolenic acid (C18:3), the oxidizability of fats was calculated according to Equation 3 (Farmani, Hamedi, Safari, & Madadlou, 2007).

(3) $$\text{Oxidizability}=\left[\left(\%\;C18:1\times 0.02\right)+\left(\%\;C18:2\right)+\left(\%\;C18:3\times 2\right)\right]/100$$

### 2.11 Statistical methods

All shown data represent the mean values ± standard deviation of triplicate experiments analyzed using SPSS version 22.0 (SPSS Inc.). Differences among the samples were statistically analyzed using one‐way analysis of variance (ANOVA) at level of *p* < .05, followed by post hoc Duncan test.

## 3 RESULTS AND DISCUSSION

### 3.1 Fatty acid and TAG composition

Table 1 presents the fatty acid composition of base stocks and the IE samples. Palmitic (52.9%) and oleic (30.1%) acids were the most abundant fatty acids in PS, and the predominant fatty acids in SFO were linoleic acid (ω_6_ which is an essential fatty acid), oleic acid, and palmitic acid (54.62%, 32.54%, and 7.32%, respectively). All the obtained blends contained TFA <0.36%. Based on defined regulations, *trans*‐free products should have <2% of the *trans*isomer (Farmani & Gholitabar, 2015). The blends obtained in this study had lower than 0.36% TFA which was favorable. The amount of saturated fatty acids (SFAs) in blends from 10:90, 30:70, and 50:50 were 16.26%, 25.75%, and 35.26%, respectively. The high oxidative stability is one of the most important features of fats, which has a great impact on the shelf life of final products (Ghotra, Dyal, & Narine, 2002). Due to higher oxidative stability of SFAs, the higher content of them can be more desirable in fat formulations.

**Table 1:** Fatty acid composition, triacylglycerol composition, and calculated iodine value of base stocks and interesterified blends

Fatty acids (%)		Base stocks		Blends (palm stearin: sunflower oil)		
		Palm stearin	Sunflower oil	10:90	30:70	50:50
16:0		52.9 ± 0.06	7.32 ± 0.03	11.87 ± 0.04	20.97 ± 0.02	30.11 ± 0.03
18:0		6.1 ± 0.03	4.21 ± 0.01	4.38 ± 0.02	4.76 ± 0.02	5.16 ± 0.03
18:1		30.1 ± 0.05	32.54 ± 0.05	32.30 ± 0.02	31.81 ± 0.04	31.30 ± 0.05
18:2		7.8 ± 0.02	54.62 ± 0.07	49.90 ± 0.03	40.55 ± 0.02	31.22 ± 0.02
18:3		0.1 ± 0	0.20 ± 0.03	0.19 ± 0.02	0.16 ± 0.01	0.15 ± 0.03
TFA		0.6	0.2	0.16	0.26	0.36
SFA		60.8	11.53	16.26	25.75	35.26
USFA		38.60	87.56	82.55	72.78	63.03
PUFA		7.9	54.82	50.12	40.74	31.36
USFA/SFA		0.63	7.59	5.07	2.82	1.78
PUFA/SFA		0.13	4.75	3.08	1.58	0.89
Calc. IV		39.7	127.40	126.63	125.09	123.55
Triacylglycerol composition (%)[Link]						
OOO	NIE	2.00	3.00	2.90	2.70	2.5
	IE			3.37	3.21	3.06
OLO	NIE	1.00	11.00	10.00	8.00	6.00
	IE			15.61	12.30	9.17
LLL	NIE	0	27.20	24.48	19.04	13.60
	IE			12.42	6.66	3.04
LOL	NIE	3.00	29.20	26.58	21.34	16.10
	IE			24.12	15.69	9.15
POO	NIE	17.50	3.50	4.90	7.70	10.50
	IE			3.71	6.37	8.84
SOO	NIE	1.00	1.10	1.09	1.07	1.05
	IE			1.37	1.45	1.51
PLL	NIE	0.70	9.60	8.78	9.93	5.15
	IE			8.86	10.35	8.80
PLO	NIE	5.00	10.00	9.50	8.50	7.50
	IE			11.48	16.23	17.65
POP	NIE	33.50	0.50	3.80	10.40	17.00
	IE			1.36	4.20	8.51
POS	NIE	5.50	0.40	0.91	1.93	2.95
	IE			1.00	1.91	2.91
PLP	NIE	7.50	0.60	1.29	2.67	4.05
	IE			2.10	5.35	8.49
PPP	NIE	18.70	0.80	2.59	6.17	9.75
	IE			0.16	0.92	2.73
PPS	NIE	3.60	0.40	0.72	1.36	2.00
	IE			0.18	0.63	1.40
S3	NIE	22.90	1.20	3.37	7.71	12.05
	IE			0.35	1.55	4.13
U3	NIE	6.00	70.40	63.96	51.08	38.02
	IE			55.92	37.96	24.48
U2S	NIE	24.20	24.20	24.20	24.20	24.20
	IE			25.40	34.48	36.90
S2U	NIE	46.50	1.50	6.00	15.00	24.00
	IE			4.49	11.49	19.96

Note

Values are shown as mean ± standard deviation.

Abbreviations: Calc. IV, calculated iodine value; IE, interesterified; NIE, noninteresterified; PUFA, polyunsaturated fatty acids (sum of C18:2 and C18:3); S2U, disaturated–monounsaturated; S3, trisaturated; SFA, saturated fatty acids (sum of C12:0, C14:0, C16:0, and C18:0); TFA, *trans*fatty acids; U2S, monosaturated–diunsaturated; U3, triunsaturated; USFA, unsaturated fatty acids (sum of C18:1, C18:2, C18:3, and TFA).

TAG composition of palm stearin and sunflower oil was obtained from Costales‐Rodríguez et al. (2009) and Lida et al. (2002). TAG composition of the IE blends was calculated based on the 1, 2, 3‐random theory following the probability low.

As can be seen in Table 1, adding SFO to PS increased the content of polyunsaturated fatty acids (PUFAs). This result indicated that using such blends could be appropriate for healthy fats. The oleic acid and linoleic acid levels of the blends were more than 31.30% and 31.22%, respectively, while the linolenic acid content was <0.19%. Due to low oxidative stability of linolenic acid, its high content in fat formulations accelerates the decay, thereby causing off‐flavors, toxic compounds, loss of nutritional value, and unusable fat products. The toxic compounds resulting from rancidity can cause problems such as tumors, heart failure, cataract, and brain dysfunction (O'Brien, 2008). The predominant SFA of the blends was palmitic acid (11.87%–30.11%), which imparts a desirable smooth consistency required for producing plastic fatty products. As reported in previous researches, fatty acid composition can affect the fat crystal habit (crystal form of TAG). Fats with higher palmitic acid content are more stable in *β*′‐crystal form than those with less palmitic acid. The *β*′‐crystal form of TAG promotes a desirable smooth consistency and plasticity required for plastic fat products (Jeyarani & Yella Reddy, 2003; Soares et al., 2012).

According to published studies, the interesterification does not affect TFA content, nor the degree of saturation (Karabulut et al., 2004; Noor Lida, Sundram, Siew, Aminah, & Mamot, 2002).

In chemical interesterification, fatty acids are randomly rearranged in TAG structures without changing the fatty acid profile, until equilibrium point of reaction is reached. Accordingly, the TAG profile of IE blends can be calculated by probability laws from their fatty acid composition (Naeli, Farmani, & Zargarran, 2016; Saghafi et al., 2018). The calculated TAG composition of NIE and IN blends is presented in Table 1. In NEI blends with 10% or 30% PS, the dominant TAGs were LLL (24.48% and 19.04%) and LOL (26.58% and 21.34%), while the major TAGs of 50:50 NIE blend was POP (17.00%) and LOL (16.10%). After chemical interesterification, the content of OOO, OLO, SOO, PLL, PLO, POP, and PLP increased but LLL, LOL, POO, POP, and PPP decreased. Generally, interesterification caused a decrease in trisaturated (S3), triunsaturated (U3), and disaturated–monounsaturated (S2U) TAGs and an increase in diunsaturated–monosaturated (U2S). This finding was also reported by Costales‐Rodríguez et al. (2009) and Naeli et al., (2017).

### 3.2 Influence of interesterification on the chemical properties of final blends

IV is an indicator for assessing the degree of unsaturation in oils and fats (Taghvaei et al., 2013). As expected, IV increased with increasing SFO ratio, as shown in Table 1. Results of FFA, PV, and soap content of the blends are presented in Table 2.

**Table 2:** Changes in peroxide value, free fatty acids, and soap content of the fat blends after interesterification and oxidizability of initial blends

Stock/blend	FFA (%)		PV (meq/kg)		Soap content (ppm)		Oxidizability
	NIE	IE	NIE	IE	NIE	IE	
Base stocks							
Sunflower oil	0.04 ± 0.003	ND	1.1 ± 0.02	ND	0	ND	0.556
Palm stearin	0.03 ± 0.002	ND	5.5 ± 0.04	ND	0	ND	0.086
Blends (Palm stearin: Sunflower oil)							
10:90	0.04 ± 0.005^b^	0.16 ± 0.032^a^	2.0 ± 0.01^a^	0.9 ± 0.1^b^	0	19.43 ± 3.7	0.509
30:70	0.05 ± 0.003^b^	0.13 ± 0.011^a^	2.6 ± 0.03^a^	1.0 ± 0.2^b^	0	22.83 ± 4.2	0.415
50:50	0.04 ± 0.012^b^	0.10 ± 0.024^a^	3.7 ± 0.04^a^	0.6 ± 0.3^b^	0	19.78 ± 2.8	0.321

Note

Mean (*n = 3*) values ± *SD* with different lower case letters represents the significant effect of interesterification in the same blend at *p* < .05.

Abbreviations: FFA, free fatty acids; IE, interesterified; ND, not determined; NIE, noninteresterified; PV, peroxide value.

The storage quality of oils is predicted by their FFA content. It causes off‐flavor extension in oils during storage (O'Brien, 2008). FFAs impair catalyst performance, and their level in initial blends should be maintained as low as possible, preferably below 0.1%. As can be seen from Table 2, chemical interesterification led to a significant increase in FFA % (*p* < .05). The FFA content of the interesterified PS: SFO blends (0.10%–0.16%) was much less than that reported by Farmani et al. (2009) (0.81%–1.08%) and Kowalski, Tarnowska, Gruczynska, and Bekas (2004) (0.9%–2.9%). The lower the FFA content, the higher the oxidative stability of fats. Petrauskaite, De Greyt, Kellens, and Huyghebaert (1998) reported that good catalyst activity results in a clear increase in FFA. Farmani et al. (2009) also reported a significant increase in FFA content of canola oil/palm olein or fully hydrogenated soybean oil blends after both chemical and enzymatic interesterification. So far, no clear reason for this has been reported. However, this may be due to the catalyst (sodium methoxide) mechanism. In sequence, the ester bonds of fatty acids and glycerol backbone; then, the newly liberated fatty acids are randomly shuffled within a fatty acid pool and re‐esterified onto a new position, either on the same glycerol (intraesterification) or onto another glycerol (interesterification). This mechanism continues until the reaction is stopped (Akoh & Min, 2008). In fact, the separation and rearrangement of fatty acids can be the reason for a slight increase in FFAs after intraesterification.

Peroxide value is an indicative of primary oxidation compounds. Although the primary oxidation compounds have no odor, carbonyl compounds formed by their decomposition are odoriferous (O'Brien, 2008).

According to Table 2, PV of all the blends decreased significantly after chemical interesterification (*p* < .05). The absorption of peroxides onto soaps during chemical interesterification could be probably the main reason for lower PV values after interesterification. The PV of initial blends increased with increasing proportion of PS, due to the higher PV of PS (5.5 meq/kg).

Soap is produced during chemical interesterification due to the alkaline nature of sodium methoxide and the presence of water added to inactivate the catalyst. The noninteresterified (NIE) blends were soap‐free, while the soap content in the IE samples was 19.43%–22.83% (Table 2). Soap is the main byproduct of interesterification, which is produced during catalyst inactivation. The soaps produce FFAs during interesterification especially in the catalyst inactivation step (Petrauskaite et al., 1998). Although postbleaching could greatly reduce the soap content, to remove it completely, washing with warm water is necessary. Therefore, postbleaching and deodorization processes are necessary for the removal of impurities (soap and free fatty acids) from chemically IE fats.

### 3.3 Oxidative stability of final blends after interesterification

**Figure 1:**
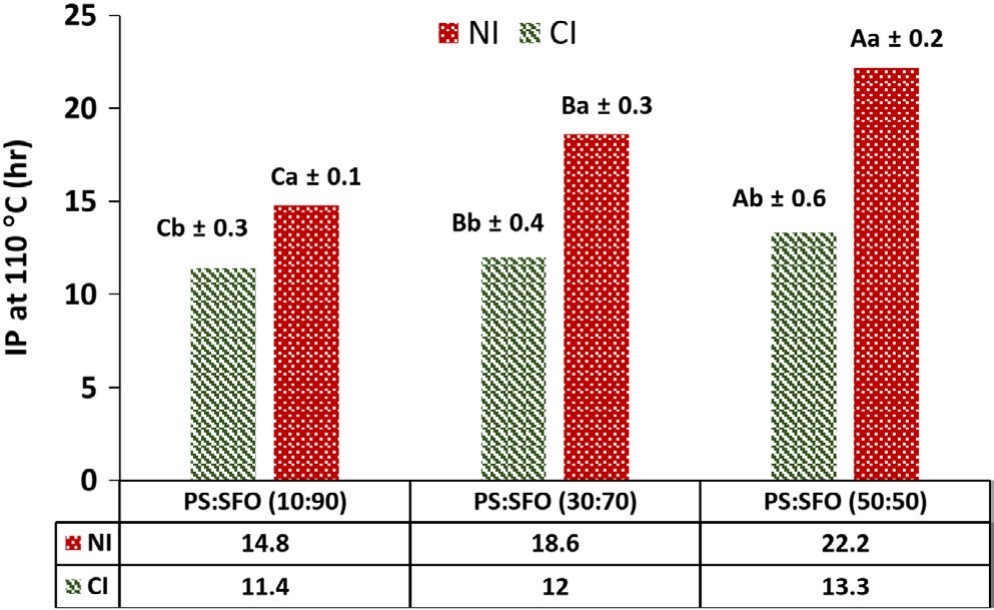
Effect of interesterification on induction periods at 110°C (IP110) of palm stearin: sunflower oil blends. Mean (*n = 3*) values ± *SD* with different lower case letters represents the significant effect of interesterification in the same blend, and values with different capital letters represent the significant effect of proportion of palm stearin in blend at *p* < .05. IE, interesterified; NI, noninteresterified; PS, palm stearin; SFO, sunflower oil

As can be seen in Table 2, PS had a very low oxidizability (0.086) due to containing more than 60.8% SFA (Table 1), while oxidizability of SFO was relatively high (0.556). So, following the increase in the PS proportion within the blends, oxidizability of was decreased. By increasing PS in the formulation, oxidative stability of the fat blends was also increased (*p* < .05). Figure 1 shows the IP_110_ of initial and IE samples. As shown, with the increase in PS content, IP_110_ increased more. Our results revealed that IP_110_ of IE blends was reduced (3.4–8.9 hr) compared with the initial blends (14.8–22.2 hr). In other words, our data showed a significant decrease in oxidative stability of the IE blends after interesterification (*p* < .05).

As can be seen in Table 2 and Figure 1, there was a negative correlation between oxidizability and IP_110_. According to previous studies, the decrease in oxidation stability of IE fats is mainly due to redistribution of polyunsaturated fatty acids from the glycerol's internal position (*β* position) onto the external positions (*α* and *α*′ positions) caused by chemical interesterification (Wang, Jiang, & Hammond, 2005). In fact, the oxidation rate of blends was increased after interesterification, even though, the PV decreased by this process. It can be summarized that the increase in polyunsaturated fatty acids connected in the external positions of TAG molecules is the main reason for the decrease in oxidative stability of fat blends by chemical interesterification.

### 3.4 Influence of interesterification on the physical properties of final blends

The SMP of initial and IE blends are presented in Table 3. The SMP of NIE blends increased with the addition of PS as a result of the higher content of trisaturated TAGs. The SMP of IE blends was lower than their initial samples (*p* < .05).

**Table 3:** Changes in slip melting point and plastic range of palm stearin and sunflower oil blends after interesterification

Blends	SMP (°C)		Plastic range (°C)	
	NIE	IE	NIE	IE
PS:SFO				
10:90	34.1 ± 0.7^a^	25.9 ± 0.9^b^	NIR (<5)	NIR (<5)
30:70	40.6 ± 0.4^a^	31.3 ± 1.1^b^	10.7–16.0	7–11.0
50:50	45.2 ± 0.8^a^	36.7 ± 0.9^b^	25.2–34.5	19.0–28.3

Note

Mean (*n = 3*) values ± *SD* with different lower case letters represents the significant effect of interesterification in the same blend at *p* < .05.

Abbreviations: IE, interesterified; NIE, noninteresterified; NIR, not plastic in SFC measurement range; PS, palm stearin; SFO, sunflower oil; SMP, slip melting point.

**Figure 2:**
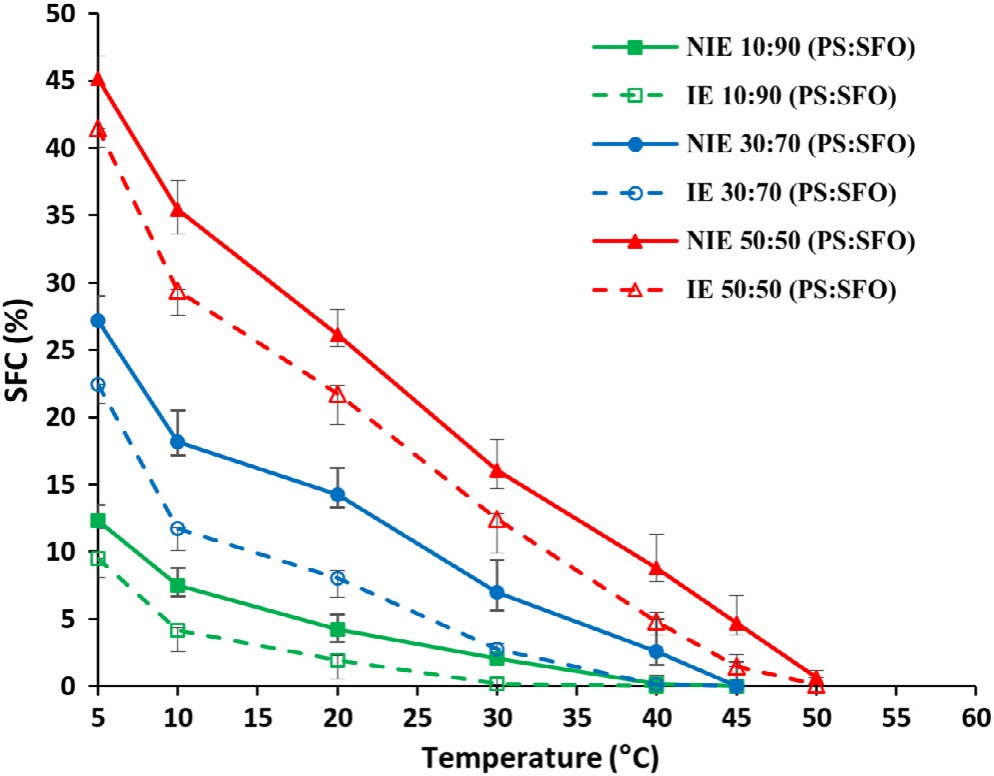
The solid fat content (SFC) curves of palm stearin: sunflower oil blends before and after interesterification. The mean (*n = *3) SFC of blends is shown at each temperature, and *error bars* show the range of each point. IE, interesterified; NIE, noninteresterified; PS, palm stearin; SFO, sunflower oil

The SFC curves of interesterified and physical blends are illustrated in Figure 2. Both temperature and the proportion of PS directly affected the rate of SFC evolution. The SFC increase at higher ratio of PS in the blends may be due to the content of SFA (16.26–35.26, Table 1) and high‐melting trisaturated TAGs (*p* < .05). The IE blends showed a reduced SFC almost at all measured temperatures (Figure 2, *p* < .05).

Plastic range is the temperature ranges in which the fats have a plastic form (Metzroth, 2005). As can be seen in Table 3, with the increase of PS in blends, plastic range transferred to the higher temperatures. The 10:90 NIE and IE blends were plastic at temperatures higher than 5°C, which was not in the range of SFC measurement temperature. After interesterification, the plastic range transferred to lower temperatures as result of decreasing the SFC content (Table 3).

In general, SMP and SFC of the fat blends decreased after interesterification (Table 3 and Figure 2). Other studies have also reported similar results to those of this study (Costales‐Rodríguez et al., 2009; Soares et al., 2012). After interesterification, the high‐melting trisaturated TAGs decrease, while the disaturated–monounsaturated and monosaturated–diunsaturated TAGs content increase. It can be considered as the main reason of SMP and SFC reduction by interesterification (Soares et al., 2012). The SMP of blends reduced up to 8.2–9.2°C after interesterification. Fatty bases for producing margarine should completely melt at body temperature to eliminate waxy mouthfeel (Kim, Lumor, & Akoh, 2008). The SMPs of the IE blends were between 25.9 and 36.7°C, indicating that the IE samples melt approximately at body temperature.

As already noted, the SFCs of IE blends were lower than their initial ones, indicating that the IE blends became more fluid. The SFC above 4% causes a waxy mouthfeel; therefore, the SFC reductions during interesterification are positive points in sensory characteristics of fat products (Masuchi et al., 2014). The SFC at 10°C is an indicator for assessing the spreadability of the fats at low temperatures. A SFC <32% (at 10°C) is necessary for good spreadability (da Silva et al., 2010). The SFC of the 10:90, 30:70, and 50:50 IE blends at 10°C were 4.16%, 11.70%, and 29.41%, respectively, which indicate these blends may have a good spreadability at refrigeration temperatures.

As discussed above, IE blends had a lower plastic range than initial blended samples. In fact, the IE blends had lower contents of SFAs and trisaturated TAGs and, consequently, lower SFC and plastic range than NIE blends.

### 3.5 Potential applications of the interesterified palm stearin: sunflower oil blends

**Figure 3:**
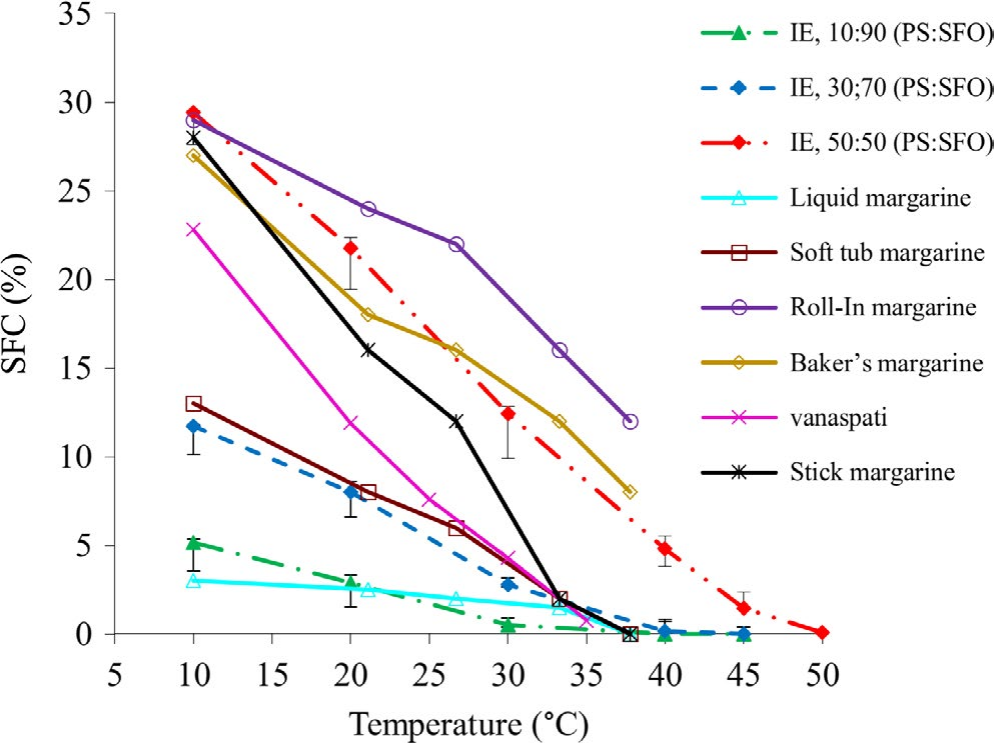
The collation solid fat content (SFC) curves of the interesterified (IE) palm stearin (PS): sunflower oil (SFO) blends versus some plastic fats

Chemical interesterification is used in the food industry to produce *trans*‐free margarines and shortenings. One of the main physical features of fats is their SFC, which correlates with some functional properties such as consistency, spreadability, and sensorial acceptance. The SFC curve can be used to determine the special applications of fats (Augusto, Soares, Chiu, & Gonçalves, 2012; Ghotra et al., 2002). Therefore, the SFC curves of the IE PS: SFO fats were compared with the common SFC curve of different margarines to evaluate their potential applications for different products (Figure 3). Studying the similarity of SFC curve of each blend to the conventional SFC curve of any type of fats can show its potential.

Recently, there is a constantly increasing demand for the production of liquid margarine, because of its high pump‐ability, ease of use and storage, and low saturation (Ronzio, 2003). As shown in Figure 3, the SFC curve (melting behavior) of 10:90 IE blend is similar to that of the liquid margarine. As a result, 10:90 IE blend can be a good option for liquid margarine production. The spreadability of soft‐tub margarines at low temperatures is a key factor in their acceptability. Therefore, this type of margarines should not be very saturated (O'Brien, 2008). As shown in Figure 3, the IE blend containing 30% PS and 70% SFO was suitable for soft‐tub margarine production. Commercial soft‐tub margarine has a melting point of 33.3°C, which is equal to the SMP of 30:70 IE blends (Ghotra et al., 2002).

Stick margarine contains higher saturated fat compared with soft‐tub margarine or liquid margarine and has a stiffer consistency than shortening. The SFCs of 28% (at 10°C), 16% (at 21.1°C), 12% (at 26.7°C), 2% (at 33.3°C), and 0% (at 37.8°C) have been reported for typical stick margarines (Ghotra et al., 2002). It seems an IE blend containing 30%–50% PS and 50%–70% SFO has the potential to be used as the basis‐stock of stick margarine (Figure 3).

Roll‐in margarine is also widely used in the production of puff pastry (such as Danish pastry). Pastry fats are very structured fats along with a crystal matrix, providing required stretching characteristic yet retain moisture when dough faced to extrusion. The shear force causes extrusion to break fat/water emulsions. This fat is spread between sheets of dough in puff pastry. The layers of dough are kept separate and flaky by fats and also the moisture which is present in the puff as it turns to steam during the baking process (Ghotra et al., 2002). As illustrated in Figure 3, the 50:50 IE blend can be suitable for roll‐in pastry applications.

Bakery fats (i.e., shortening or margarine) should provide desirable tenderness, texture, and mouthfeel and result in the extending of product shelf life. As can be seen from Figure 3, the IE 50:50 blend can be used in the production of bakery margarine (Ghotra et al., 2002).

## 4 CONCLUSION

Our study indicated that after interesterification of palm stearin and sunflower oil, FFA and soap content increased and peroxide value and IP_110_ of blends decreased. Interesterification also improved the melting properties and plastic range of the fat blends. It was revealed that the interesterified blends display the desired solid fat content (SFC) profiles, appropriate for the production of a variety of *trans*‐free fats, offering good alternatives to hydrogenated fats.

## CONFLICT OF INTEREST

All authors declare that there is no conflict of interest.

## ETHICAL APPROVAL

There was no human or animal testing in this study.
